# Factors Influencing Home Death in a Japanese Metropolitan Region

**DOI:** 10.4061/2011/610520

**Published:** 2011-05-29

**Authors:** Akiko Akiyama, Hiroo Hanabusa, Hiroshi Mikami

**Affiliations:** ^1^Division of Health Sciences, Osaka University Graduate School of Medicine, Osaka 565-0871, Japan; ^2^Shinjuku Hiro Clinic, Shinjuku-Ward, Tokyo 160-0023, Japan

## Abstract

To examine factors influencing home death, an anonymous survey was mailed to 998 home care supporting clinics (HCSCs) in the 23 wards of Tokyo, Japan. We classified the HCSCs into two types (single physician practice and multiple physician practice) and identified factors of each type of practice that predict home death. The factors associated with a greater probability of dying at home were as follows: in the multiple physician practices, collaboration with hospitals and teaching coping skills to the family members and, in the single physician practices, collaboration with clinics. Our findings suggest that home end-of-life care services are unlikely to be achieved without cooperation among service providers and without improvement of the family members' coping skills.

## 1. Introduction

For Japanese people the preferred place of death has usually been home [1, 2]. A 12.3% of home death has been recorded for the year 2007 [3]. Miyata et al. suggested that end-of-life care and home death was not a very practical option in Japan because the quality of home care was not satisfactory until recently [4]. Previous studies have also observed that patients prefer end-of-life and death to happen at home; however this preference is not often recorded as an actual place of death [5–9]. Steinhauser et al. have indicated that many people prefer to die at home, but, primarily, there are other important factors which need to be addressed before consideration of home death including pain and symptom management, preparation for death, achieving a sense of completion, decisions about treatment preferences, and being treated as a “whole person” [8]. Beccaro et al. emphasized that policy makers should encourage health services to focus on ways of meeting individual preferred places of death [9].

Previous studies have found that certain features of the home care system are associated with place of death [10–13]. For instance, home visit by general practitioners (GPs) is a factor that contributes to high incidence of home death [10]. Fukui et al. [11] reported that the number of home visits per week by home care nurses influenced the incidence of home death. Grande et al. [12] reported that the commonly mentioned factors in care evaluations by GPs and district nurses were their accessibility, enlistment of support from other agencies, and their ability to ensure the availability of equipment and supplies. Rosenquist et al. [13] mentioned that a key factor for the success of home care is the availability of a GP and nurses, as well as an access to hospital bed as and when required, and emphasized that these factors need to be satisfied before considering home death.

**Figure 1:**
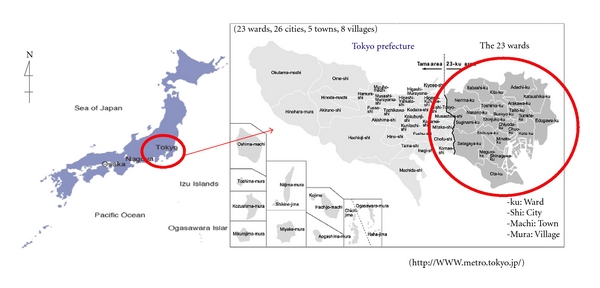
The location of the 23 wards of Tokyo metropolitan region in Japan.

Some studies have reported that home death is also influenced by the geographical locations of patient's residence, because the home care system differs in the metropolitan and the rural areas [14–16]. Gomes and Higginson [14] state that patients in rural areas are more likely to die at home because they have difficulties in accessing health care. Houttekier et al. [15] suggested that metropolitan patients were less likely to die at home because of poor social support and a lower availability of home care beds. In the 23 wards of the Tokyo metropolitan region, a population density of 14,153 people per square kilometer was recorded for the year 2010 [17] (Figure 1), and the home care supporting clinics (HCSCs) in the 23 wards of Tokyo are easily accessible. However, the types of home care systems that enable home death have not been studied specifically in a metropolitan setting.

Considering the proportion of aged people population in Japan, there is an urgent need for providing the provisions of medical and end-of-life care that are available in hospitals to homes [18]. Japanese long-term care insurance was introduced in 2000 to promote the socialization of care for frail elderly [19]. The Japanese Cancer Control Act was implemented in April 2007 [20]. Palliative care from the early phase of treatment is one of its basic concepts, which address home-based palliative care that enables cancer patients to spend their end-of-life period and to die at home, considering that as few as 6.7% cases of home deaths have been recorded for cancer patients in 2007 [3]. While palliative care units have been covered by the National Medical Insurance since 1990, home-based palliative care has only been covered recently in 2002.

With this background, Japanese HCSCs were newly introduced by the revised Medical Care Act in April 2006 [21]. HCSCs are expected to play a central role in the provision of end-of-life care at home by providing home care services 24 hours a day and by cooperating with hospitals, home-visit nursing stations, and care managers and ensuring emergency hospital admission. The number of HCSCs in Japan is rapidly increasing. It amounted to 11,539 as of September 2010; in particular, those in the 23 wards of the Tokyo metropolitan region account for approximately 10% of all HCSCs in Japan [22]. However, the activities conducted at HCSCs are not altogether clear because statistical data regarding the activities of HCSCs and the actual operating system have not been disclosed to the public. Thus, the contribution of home care system practiced by HCSCs in influencing the choice of the place of death is still unclear.

Therefore, the purpose of this study was to determine the influence of the home care system practiced by HCSCs in the Tokyo metropolitan region on home death and to identify features of single and multiple physician practices that influence home death. 

## 2. Methods

The objects of this study were 998 clinics in the 23 wards of Tokyo, Japan that were certified as HCSCs by the Japanese Ministry of Health, Labor, and Welfare as of March 1, 2009. A self-administered questionnaire was mailed in collaboration with the Japan Network of Home Care Supporting Clinics [21] during July 2009 to August 2009. This survey protocol was approved by the Ethics Committee of the Department of Medicine, Osaka University.

We, in the questionnaire, queried the clinic's characteristics, collaboration with other agencies (hospital, clinic, home visit nursing station, and care manager), the number of patients, and home care self-assessments.

Home care self-assessment was developed on the basis of our previous study [23]. Representative individuals of the clinics self-rated their activities on behalf of the facility on a scale of 1–5 (“strongly disagree” to “strongly agree”). Each HCSC was classified into two types by the number of physicians engaged in the practice: (1) single physician practice (single) and (2) multiple physician practice (multiple). Student's *t*-test, Fisher's exact test, and Mann-Whitney's *U*-test were used to compare the differences according to the number of physicians.

To examine the relationship between the characteristics of HCSCs and the proportion of home deaths, we further classified HCSCs into two groups by the proportion of home deaths: (1) less than 10% (<10%) and (2) equal to and more than 10% (≥10%). In this analysis, we excluded the clinics with the following features: (1) those where the number of total patients was less than 10 persons per year; (2) those where the number of total patients or patients who died at home was unclear. We then compared the differences according to the proportion of home deaths using Student's *t*-test, Fisher's exact test, and Mann-Whitney's *U*-test. Next, we performed stepwise multiple linear regression analysis using items that were significant in Student's *t*-test, Fisher's exact test, and Mann-Whitney's *U*-test as dependent variables. Statistical analysis was performed using SPSS 12.0 J for Windows. The level of significance was set at *P* < .05.

## 3. Results

Out of the 998 clinics in the 23 wards, only 994 clinics could be contacted. We received 183 responses (response rate: 18.4%) and 166 were finally analyzed; 17 responses were excluded because they were incompletely answered (effective response rate: 16.6%).

Table 1 shows a comparison of characteristics between the single and multiple physician practices. The multiple physician practices employed significantly more health workers (including nurse, social worker, and others) and had sufficient medical care equipments such as ventilators and IVH. In addition, they operated significantly more often in parallel with other institutions including hospitals, specific facilities, home visit nursing stations, and home help services and also collaborated significantly more often with hospitals, clinics, home visit nursing stations, and care managers.

Table 2 represents the number of patients. Among 15,027 patients referred to 166 HCSCs in 2008, 1083 died at home (home death rate: 12.3%). Thirty-nine clinics, 86.4% of which were single physician practices, had no patients with home death case. The total number of patients, the number of patients with home death, and the number of patients living alone were significantly higher for multiple physician practices. There were no significant differences in the total numbers of patients per physician and patients who died at home per physician between the two groups. The patients who were living alone and died at home were significantly more frequently provided home care by multiple physician practices.

Tables 3 and 4 show the relationship between the characteristics of HCSCs and the proportion of home deaths. Single physician practices with ≥10% of home deaths significantly collaborated with other clinics and rated themselves high on the factor that the patients could be admitted to hospitals when symptoms were aggravated. Multiple physician practices with ≥10% of home deaths significantly collaborated with hospitals and rated themselves high on these factors: that the patients could be admitted to hospitals in case of emergency, that service use was available when required without delay, that the physician provided sufficient explanations to families regarding the present patient's condition and the details of their medical treatment, and that the physician or nurse taught the family members coping skills for medical procedures and nursing care skills.

Table 5 shows the relationship between the characteristics of HCSCs and the proportion of home deaths. Factors enabling an increase in the proportion of home deaths were as follows: collaboration with clinics (*β*: 0.33) in case of the single physician practices; collaboration with hospitals (*β*: 0.37) and the physician or nurse teaching the family members coping skills in case of medical procedures and nursing skills to take care of the patient (*β*: 0.33) in the multiple physician practice. 

## 4. Discussion

We conducted the present study to evaluate the influence of home care systems on the incidence of home death. There are several key findings.

First, our results suggest that teaching the family members coping skills in case of medical procedures and nursing skills to take care of the patient may be the factors influencing an increased preference for home death. Previous studies indicated that the choice of the place of death is strongly influenced by the psychological condition of the caregiver [24, 25]. Recent studies demonstrated that interventions to improve the coping skills of caregivers were effective for promoting their psychological well-being of the caregiver [26–28]. Considering that the family members' concerns about the patient's condition can be eased by assisting them in providing personal care to the patient, it appears that improvement in the coping skills of the family members leads to increased preferences of spending end-of-life period and dying at home by the patient.

Second, we found that collaboration with hospitals was associated with a greater probability of home death preferences in multiple physician practices. Hospitals have been requested by the Japanese Medical Care Law to collaborate with clinics for providing continual patient care [29]. Taniguchi reported that GPs are strongly concerned with the availability of emergency hospitalization facilities [30]. The findings suggest that, for continual patient care, it is important to establish a cooperative structure between hospitals and clinics. In addition, in the single physician practices also, collaboration with other clinics was an important factor influencing home deaths. For single physician practice HCSCs, the provision of home care services 24 hours a day was a challenging task, especially during out-of-hours [31, 32]. Thus, some of these practices have initiated a new approach to improve the function of HCSCs [33, 34]. For example, some groups of HCSCs constructed a network among themselves and conducted out-of-hours home care services on a rotation basis [33]. Under these circumstances, the provision of the option of end-of-life care and home death to the patient would become possible.

Third, our findings indicated that the multiple physician practices enabled continuous home care and dying at home for various patients. They had several advantages as follows: (1) larger number of physicians and health workers, (2) sufficient medical care equipments, and (3) more collaboration with other agencies. Such types of HCSCs are fewer in sparsely populated rural areas owing to the difficulty in efficient management of such institutions compared with clinics in the 23 wards of the Tokyo metropolitan region. [17]. Previous studies have reported that successful home care depends on their availability and accessibility [13]; our results showed that the multiple physician practice HCSCs in the 23 wards of Tokyo are located close to residents of the patients and have various resources for home care.

In agreement with previous studies, the 23 wards of the Tokyo metropolitan region have few incidences of home deaths [35]. Hence, there is an urgent need to improve the home care systems in the 23 wards of Tokyo because of the increase in the proportion of aged people population in Japan [36]. Our study identified valuable factors that influence rate of home death in the 23 wards of Tokyo.

This study had several limitations. First, the response rate was only 18.4%. The objects of our study were registered clinics such as the HCSCs but we were not able to identify functional clinics among them because any clinic which meets the requirement set under the Japanese Ministry of Health, Labor, and Welfare can acquire a certification of a HCSC. Therefore, we suspect that a considerable number of nonfunctioning HCSCs did not respond to our survey. However, it is actually unclear how the HCSCs are operated under the system because statistical data regarding the activities of HCSCs have not been disclosed to the public in Japan. Therefore, we believe that our findings provide a basis to examine the home care system of HCSCs that enable spending end-of-life period and home death. Second, we classified the HCSCs into two types on the basis of the number of physicians and compared differences between these two groups; however, there are other criteria which were not considered such as the type of clinic and type of management practiced. In future, the association of home death with the type of clinic should be studied.

In conclusion, our findings indicated that home care services in the metropolitan region are unlikely to be achieved without cooperation of service providers and without improvement of family coping skills. 

## Figures and Tables

**Table 1:** Characteristic of the HCSCs.

	Total	No. clinics (%)		*P* value
	*n* = 166	Single, *n* = 91	Multiple, *n* = 75	
Health workers				
Physicians^a^	2.6 ± 3.3	1	4.6 ± 4.1	<.001
Nurses^a^	2.7 ± 3.7	1.4 ± 1.3	4.1 ± 4.8	<.001
Social worker (Yes)	17 (10.2)	1 (1.1)	16 (21.3)	<.001
Others (Yes)	64 (38.6)	21 (23.1)	43 (57.3)	<.001
Providing medical care				
Oxygen inhalation	146 (88.0)	77 (84.6)	69 (92.0)	.228
Ventilator	63 (38.0)	27 (29.7)	36 (48.0)	.024
Intravenous hyperalimentation (IVH)	106 (63.9)	50 (55.6)	56 (74.7)	.014
Percutaneous endoscopic gastrostomy (PEG)	110 (66.3)	56 (62.2)	54 (72.0)	.246
Palliative medicine	119 (71.7)	60 (66.7)	59 (78.7)	.116
Type of clinics				
Single	113 (68.1)	77 (84.6)	36 (48.0)	<.001
Multiple (established other institutions in parallel)^b^	41 (24.7)	10 (11.0)	31 (41.3)	
Hospital	9 (5.4)	1 (1.1)	8 (10.7)	.004
Specific facility	7 (4.2)	1 (1.1)	6 (8.0)	.048
Home visit nursing station	16 (9.6)	0 0.0	16 (21.3)	<.001
Home help services	32 (19.3)	7 (7.7)	25 (33.3)	<.001
Collaboration with other agencies^c^				
Hospital				
0	5 (3.0)	3 (3.3)	2 (2.7)	<.001
1	47 (28.3)	33 (36.3)	14 (18.7)	
2	32 (19.3)	21 (23.1)	11 (14.7)	
3	29 (17.5)	18 (19.8)	11 (14.7)	
≥4	49 (29.5)	15 (16.5)	34 (45.3)	
Clinic				
0	59 (35.5)	34 (37.4)	25 (33.3)	.020
1	41 (24.7)	23 (25.3)	18 (24.0)	
2	22 (13.3)	15 (16.5)	7 (9.3)	
3	14 (8.4)	6 (6.6)	8 (10.7)	
≥4	22 (13.3)	9 (9.9)	13 (17.3)	
Home visit nursing station				
0	14 (8.4)	9 (9.9)	5 (6.7)	.002
1	29 (17.5)	17 (18.7)	12 (16.0)	
2	27 (16.3)	20 (22.0)	7 (9.3)	
3	29 (17.5)	18 (19.8)	11 (14.7)	
≥4	63 (38.0)	26 (28.6)	37 (49.3)	
Care manager				
0	58 (34.9)	40 (44.0)	18 (24.0)	<.001
1	3 (1.8)	2 (2.2)	1 (1.3)	
2	11 (6.6)	6 (6.6)	5 (6.7)	
3	7 (4.2)	5 (5.5)	2 (2.7)	
≥4	79 (47.6)	35 (38.5)	44 (58.7)	
Time taken to visit the patient's home (minutes)^c^				
≤15	65 (39.2)	40 (44.0)	25 (33.3)	.029
6–30	80 (48.2)	43 (47.3)	37 (49.3)	
1–45	13 (7.8)	4 (4.4)	9 (12.0)	
6-60	3 (1.8)	1 (1.1)	2 (2.7)	

Fisher exact test; ^a^mean ± SD; Student's *t*-test; ^b^multiple answers allowed, %; ^c^Mann-Whitney *U*-test.

**Table 2:** The number of patients.

	Total,	No. patients (%)		*P* value
	*n* = 166	Single, *n* = 91	Multiple, *n* = 75	
Total patients	15027 (100.0)	2105 (14.0)	12922 (86.0)	.001
Total patients per one clinic^a^	98.9 ± 277.6 (0–2561)	25.1 ± 39.8 (0–229)	192.9 ± 397.7 (0–2561)	
Total patients per one physician^a^	35.0 ± 75.4 (0–640)	25.1 ± 39.8 (0–228)	47.4 ± 103.2 (0–640)	.098
Patients who died at home	1083 (100.0)	221 (20.4)	863 (79.6)	<.001
Patients who died at home per one clinic^a^	7.0 ± 16.0 (0–161)	2.5 ± 4.5 (0–27)	12.7 ± 22.5 (0–161)	
Patients who died at home per one physician^a^	2.6 ± 4.2 (0–27)	2.5 ± 4.5 (0–27)	2.8 ± 3.7 (0–20)	.730
Patients living alone	11.4 ± 36.2 (0–370)	2.9 ± 6,9 (0–50)	22.9 ± 53.1 (0–370)	.005
Patients living alone who died at home (yes)	38 (25.7)	15 (17.6)	23 (36.5)	.013
Home death rate, %^a,b^	12.3 ± 16.0	12.3 ± 24.0	12.2 ± 14.2	.985

Student's *t*-test, ^a^mean ± SD (range), ^b^(patients who died at home/total patients) ∗ 100.

**Table 3:** Relationship between the proportion of home deaths and characteristics of the HCSCs.

	No. clinics (%)		*P* value	No. clinics (%)		*P* value
	Single, *n* = 49*			Multiple, *n* = 57*		
	<10%, *n* = 28	10%≤, *n* = 21		<10%, *n* = 35	10%≤, *n* = 22	
Health workers						
Physicians^a^	1	1		5.0 ± 4.9 (2–15)	4.8 ± 3.6 (2–20)	.871
Nurses^a^	1.0 ± 0.9 (0–5)	1.6 ± 1.4 (0–3)	.089	5.1 ± 7.5 (0–12)	3.2 ± 2.8 (0–36)	.177
Social worker (Yes)	0 (0.0)	0 (0.0)	.524	6 (19.4)	6 (27.3)	.362
Others (Yes)	6 (26.1)	7 (36.8)	.516	22 (68.8)	14 (70.0)	1.000
Providing medical care						
Oxygen inhalation	25 (89.3)	19 (90.5)	1.000	33 (94.3)	22 (100.0)	.518
Ventilator	11 (39.3)	7 (33.3)	.769	18 (51.4)	12 (54.5)	1.000
Intravenous hyperalimentation (IVH)	16 (57.1)	15 (71.4)	.377	27 (77.1)	20 (90.9)	.287
Percutaneous endoscopic gastrostomy (PEG)	22 (78.6)	16 (76.2)	1.000	27 (77.1)	16 (72.7)	.758
Palliative medicine	19 (67.9)	16 (76.2)	.750	28 (80.0)	19 (86.4)	.725
Type of clinics						
Single	23 (82.1)	14 (77.8)	.721	18 (52.9)	10 (52.6)	1.000
Multiple (established other institutions in parallel)	5 (17.9)	4 (22.2)		16 (47.1)	25 (47.2)	
Collaboration with other agencies^b^						
Hospital						
0	1 (3.6)	0 (0.0)	.066	0 (0.0)	0 (0.0)	.016
1	13 (46.4)	2 (9.5)		10 (28.6)	2 (9.1)	
2	3 (10.7)	9 (42.9)		7 (20.0)	3 (13.6)	
3	6 (21.4)	5 (23.8)		4 (11.4)	2 (9.1)	
≧4	5 (17.9)	5 (23.8)		12 (34.3)	15 (68.2)	
Clinic						
0	11 (39.3)	3 (14.3)	.029	14 (40.0)	5 (22.7)	.113
1	8 (28.6)	5 (23.8)		7 (20.0)	5 (22.7)	
2	5 (17.9)	8 (38.1)		3 (8.6)	3 (13.6)	
3	2 (7.1)	1 (4.8)		2 (5.7)	3 (13.6)	
≧4	1 (3.6)	3 (14.3)		6 (17.1)	6 (27.3)	
Home visit nursing station						
0	2 (7.1)	1 (4.8)	.916	3 (8.6)	0 (0.0)	.577
1	5 (17.9)	3 (14.3)		5 (14.3)	2 (9.1)	
2	4 (14.3)	1 (4.8)		3 (8.6)	4 (18.2)	
3	5 (17.9)	9 (42.9)		3 (8.6)	3 (13.6)	
≧4	12 (42.9)	7 (33.3)		19 (54.3)	13 (59.1)	
Care manager						
0	9 (32.1)	3 (14.3)	.201	11 (31.4)	2 (9.1)	.060
1	0 (0.0)	1 (4.8)		0 (0.0)	1 (4.5)	
2	3 (10.7)	1 (4.8)		2 (5.7)	1 (4.5)	
3	1 (3.6)	2 (9.5)		0 (0.0)	0 (0.0)	
≧4	14 (50.0)	14 (66.7)		19 (54.3)	18 (81.8)	
Time taken to visit the patient's home (minutes)^b^						
≦15	12 (42.9)	8 (38.1)	.684	9 (25.7)	9 (40.9)	.473
16–30	14 (50.0)	11 (52.4)		21 (60.0)	8 (36.4)	
31–45	2 (7.1)	1 (4.8)		5 (14.3)	3 (13.6)	
46–60	0 (0.0)	1 (4.8)		0 (0.0)	1 (4.5)	

Fisher exact test; ^a^mean ± SD (range); Student's *t*-test, ^b^Mann-Whitney *U*-test.

*We excluded the 60 clinics where the number of total patients was less than 10 persons per year or where the number of total patients or patients who died at home was unclear.

**Table 4:** Relationship between the proportion of home deaths and home care self-assessment.

Items	Single, *n* = 49*		*P* value	Multiple, *n* = 57*		*P* value
	<10%, *n* = 28	10% <, *n* = 21		<10%, *n* = 35	10% <, *n* = 22	
Our clinic has many patients who need intensive medical treatment.	3.6 ± 1.2 (2–5)	3.7 ± 0.8 (2–5)	.662	4.0 ± 1.0 (2–5)	3.7 ± 0.9 (2–5)	.223
The patient can be admitted to hospital in case of emergency.	2.8 ± 1.2 (1–5)	3.3 ± 0.9 (1–5)	.127	3.8 ± 1.1 (1–5)	3.2 ± 1.1 (1–5)	.042
The patient can be admitted to hospital in case of aggravation of symptoms.	2.6 ± 1.4 (1–5)	3.4 ± 0.9 (1–5)	.039	3.7 ± 1.1 (1–5)	3.2 ± 1.2 (1–5)	.132
Service use was possible when necessary without waiting.	3.2 ± 1.0 (1–5)	3.2 ± 1.0 (2–5)	.856	3.9 ± 0.9 (1–5)	3.4 ± 0.9 (2–5)	.028
Provision of care 24 hours a day is too heavy a task for our clinic.	3.5 ± 1.0 (2–5)	3.8 ± 0.9 (2–5)	.285	3.5 ± 1.4 (2–5)	4.0 ± 0.9 (1–5)	.180
Referral to home care appears too late to provide satisfactory care to the patient.	3.2 ± 0.8 (1–5)	3.1 ± 0.8 (2–5)	.700	3.6 ± 0.8 (2–5)	3.6 ± 0.8 (1–5)	1.000
The physicians attends a conference on treatment and nursing care of the patient to be held prior to patient's discharge	2.2 ± 1.5 (1–5)	2.3 ± 1.3 (1–5)	.812	3.5 ± 1.5 (1–5)	3.4 ± 1.2 (1–5)	.742
The physician give sufficient explanation to the family about the patient's present condition and the details of medical treatment.	4.2 ± 0.7 (3–5)	4.4 ± 0.6 (3–5)	.495	4.7 ± 0.5 (3–5)	4.3 ± 0.6 (4-5)	.017
The physicians give sufficient explanation to the family about the expected outcome	4.1 ± 0.7 (2–5)	4.4 ± 0.7 (3–5)	.273	4.6 ± 0.5 (3–5)	4.4 ± 0.6 (4-5)	.069
The physician dealt promptly with physical discomfort symptoms of the patient.	4.0 ± 0.9 (3–5)	3.9 ± 0.6 (2–5)	.450	4.4 ± 0.6 (3–5)	4.1 ± 0.6 (3–5)	.085
Consideration is given so that the patient can participate in the selection of treatment.	4.4 ± 0.6 (3–5)	4.3 ± 0.6 (3–5)	.455	4.5 ± 0.5 (4-5)	4.3 ± 0.5 (4-5)	.332
The family's wishes are respected in the selection of treatment.	4.4 ± 0.6 (3–5)	4.4 ± 0.6 (3–5)	.731	4.5 ± 0.5 (4-5)	4.4 ± 0.5 (4-5)	.456
The physician sufficiently talked with the family and the patient about the future plan.	4.3 ± 0.7 (3–5)	4.3 ± 0.7 (3–5)	.899	4.5 ± 0.5 (2–5)	4.3 ± 0.6 (4-5)	.176
The physician or the nurse teaches the family coping skills for medical procedure and nursing care to the patient.	3.8 ± 0.7 (3–5)	4.0 ± 0.6 (2–5)	.220	4.5 ± 0.5 (3–5)	4.2 ± 0.5 (4-5)	.022
The family could give direct nursing care to the patient.	3.5 ± 1.1 (2–5)	3.8 ± 0.6 (2–5)	.279	3.7 ± 0.7 (2–5)	3.5 ± 0.7 (2–5)	.299
Service use is in accordance with the wishes of the patient.	4.0 ± 0.8 (3–5)	3.9 ± 0.6 (3–5)	.714	4.1 ± 0.6 (3–5)	4.0 ± 0.6 (3–5)	.482
Service use is in accordance with the wishes of the family.	4.1 ± 0.8 (3–5)	4.0 ± 0.6 (3–5)	.502	4.1 ± 0.6 (2–5)	3.9 ± 0.6 (3–5)	.274
The physician visits bereaved families.	2.2 ± 1.3 (1–4)	2.2 ± 1.2 (1–5)	.901	2.9 ± 1.4 (1–5)	2.5 ± 1.2 (1–5)	.277
The nurse visits bereaved families.	2.9 ± 1.5 (1–5)	2.4 ± 1.2 (1–5)	.253	3.3 ± 1.3 (1–5)	2.8 ± 1.1 (1–5)	.148

mean ± SD (range); Student's *t*-test; items of home care self-assessments were answered by rating from 1 (highly disagree) to 5 (highly agree). *We excluded the 60 clinics where the number of total patients was less than 10 persons per year or where the number of total patients or patients who died at home was unclear.

**Table 5:** Factors influencing the proportion of home deaths.

Clinic type	Independent variables	*β*	*P*
Single^a^	Collaboration with clinics	0.33	.024

Multiple^b^	Collaboration with hospitals	0.37	.004
	Teaching the family coping skills for medical procedure and care	0.33	.010

Dependent variable: the proportion of home deaths.

Independent variables: items that were significant in Tables 3 and 4.

*β*: standardized partial regr. coeff.

^a^ *F* = 5.46, *P* < .024, *R* ^2^ = 0.11.

^b^ *F* = 7.12, *P* < .002, *R* ^2^ = 0.22.
